# I-*Sce*I-mediated double-strand DNA breaks stimulate efficient gene targeting in the industrial fungus *Trichoderma reesei*

**DOI:** 10.1007/s00253-015-6829-1

**Published:** 2015-08-15

**Authors:** Jean Paul Ouedraogo, Mark Arentshorst, Igor Nikolaev, Sharief Barends, Arthur F. J. Ram

**Affiliations:** Molecular Microbiology and Biotechnology, Institute of Biology Leiden, Kluyver Centre for Genomics of Industrial Fermentation, Leiden University, Sylviusweg 72, 2333 BE Leiden, The Netherlands; Dupont Industrial Biosciences, Archimedesweg 30, 2333 CN Leiden, The Netherlands

**Keywords:** Meganuclease, DNA repair, Counter selection, Targeted integration, *Hypocrea jecorina*

## Abstract

Targeted integration of expression cassettes for enzyme production in industrial microorganisms is desirable especially when enzyme variants are screened for improved enzymatic properties. However, currently used methods for targeted integration are inefficient and result in low transformation frequencies. In this study, we expressed the *Saccharomyces cerevisiae* I-*Sce*I meganuclease to generate double-strand breaks at a defined locus in the *Trichoderma reesei* genome. We showed that the double-strand DNA breaks mediated by I-*Sce*I can be efficiently repaired when an exogenous DNA cassette flanked by regions homologous to the I-*Sce*I landing locus was added during transformation. Transformation efficiencies increased approximately sixfold compared to control transformation. Analysis of the transformants obtained via I-*Sce*I-mediated gene targeting showed that about two thirds of the transformants resulted from a homologous recombination event at the predetermined locus. Counter selection of the transformants for the loss of the *pyrG* marker upon integration of the DNA cassette showed that almost all of the clones contained the cassette at the predetermined locus. Analysis of independently obtained transformants using targeted integration of a glucoamylase expression cassette demonstrated that glucoamylase production among the transformants was high and showing limited variation. In conclusion, the gene targeting system developed in this study significantly increases transformation efficiency as well as homologous recombination efficiency and omits the use of *Δku70* strains. It is also suitable for high-throughput screening of enzyme variants or gene libraries in *T. reesei*.

**Electronic supplementary material:**

The online version of this article (doi:10.1007/s00253-015-6829-1) contains supplementary material, which is available to authorized users.

## Introduction

*Trichoderma reesei* (teleomorph *Hypocrea jecorina*) can secrete large amounts of extracellular protein (up to 100 g/L), which makes it a paradigm host for homologous and heterologous protein production (Anderson et al. 2013; Schuster and Schmoll 2010). To improve production, activity or other properties of industrially interesting enzymes, both random and systematic approaches to generate enzyme variants are employed (Adrio and Demain 2014; Turner 2009). To simplify comparison among enzyme variants or different homologues expressed in filamentous fungi and, in particular *T. reesei*, it is highly desirable that in order to ensure an identical genetic environment, the corresponding DNA constructs are integrated at a defined locus in the genome. Random integration of expression cassettes often leads to significant variation in production levels caused by differences in copy number and/or sites of integration. Therefore, it has been attempted to increase the efficiency of gene targeting in *T. reesei* by several means, such as by increasing the size of the region homologous to the target locus (Catalano et al. 2011) or by developing strains that are deficient in non-homologous end joining (NHEJ) (Catalano et al. 2011; Guangtao et al. 2009). However, even though the efficiency of homologous integration events is tremendously increased in strains deficient in NHEJ, it remains highly locus-dependent (Schuster et al. 2012). Transformation frequencies with NHEJ-deficient strains are often low, making it difficult and laborious to generate enough mutants for library screening. Consequently, there is still a need to develop alternative methods for efficient gene targeting into the genome of *T. reesei* that lead to high homogeneity in protein expression among transformants. Another limiting factor associated with *T. reesei* transformation is a relative low frequency of stable, DNA integration events. A substantial number of primary transformants (30–50 %) are abortive ones, in which a plasmid is only transiently expressed and not integrated into the genome.

In this study, we present an approach that not only increases the number of stable transformants in *T. reesei* up to sixfold but also increases the efficiency of targeted integration of the gene of interest. The approach is based on stimulating the formation of a DNA double-strand break (DSB) at a specific locus of the genome, which is subsequently efficiently repaired by the receiving organism to maintain its genomic integrity and survival (Bollag et al. 1989; Szostak et al. 1983). Eukaryotic cells repair DSBs either by the NHEJ pathway or via homology directed repair (HDR) with homologous sequences flanking the DSBs or any other available homologous template, including exogenous donor sequences (Cahill et al. 2006). Genomic DSBs can occur either naturally or be induced artificially. It has been shown that DSBs can be specifically induced in eukaryotic genomes by using the yeast I-*Sce*I endonuclease (Arazoe et al. 2014; Choulika et al. 1995; Kuijpers et al. 2013; Puchta et al. 1993; Rouet et al. 1994). I-*Sce*I is a mitochondrial homing endonuclease encoded by the *Saccharomyces cerevisiase* mitochondrial genome and recognizes a 18-basepair-long DNA sequence (5′-TAGGGATAACAGGGTAAT-3′) (Monteilhet et al. 1990; Plessis et al. 1992; Fairhead and Dujon 1993). Since the recognition site is rather long and specific, I-*Sce*I recognition sites are absent in most eukaryotic genomes, including the *T. reesei* genome. Expression of the endonuclease I-*Sce*I has been previously demonstrated to efficiently induce DSBs at I-*Sce*I sites inserted at specific sites in prokaryotic cells (Meddows et al. 2005) and also in several eukaryotes, including mammalian cells (Choulika et al. 1995), plants (Puchta et al. 1993) and unicellular eukaryotes (Glover and Horn 2009). In filamentous fungi, I-*Sce*I-mediated DSBs have been recently described in *Pyricularia oryzae* (Arazoe et al. 2014), a fungus belonging to the class of *Sordariomycetes*, to which *T. reesei* also belongs*.* In the present report, we have introduced I-*Sce*I recognition sites into the genome of *T. reesei* and have investigated the effect of DSBs mediated by heterologous expression of I-*Sce*I on transformation efficiency, on frequency of targeted integration of a reporter gene and on the expression levels of the reporter protein. We have also shown that about two thirds of the transformants integrated a reporter *T. reesei* glucoamylase gene at a pre-assigned locus and expressed it uniformly.

## Materials and methods

### Strains and cultivation conditions

*T. reesei* strain RL-P37∆cbhIpyrG-26 (from DuPont Industrial Bioscience division, Leiden, The Netherlands) was used as a parental strain throughout the study. RL-P37∆cbhIpyrG-26 was developed from the wild-type *T. reesei* strain, QM6a, via several classical mutagenesis steps and was modified to delete the gene encoding cellobiohydrolase 1 (Cbh1) and inactivate the *pyr4* gene. *T. reesei* strains derived from the parental strain and used in this study are listed in Table 1. *T. reesei* strains were maintained on *Trichoderma* agar minimal medium (TrMM) containing 15 g/L KH_2_PO_4_, 5 g/L (NH_4_)_2_SO_4_, 20 g/L carbon source (glucose or lactose), 0.6 g/L MgSO_4_·7H_2_O, 0.6 g/L (mM) CaCl_2_·2H_2_O, 1 mL/L trace element solution (175 g/L C_6_H_8_O_7_, 200 g/L FeSO_4_·7H_2_O, 16 g/L ZnSO_4_·7H_2_O, 3.2 g/L CuSO_4_·5H_2_O, 1.4 g/L MnSO_4_·H_2_O and 0.8 g/L H_3_BO_3_) and 2 % agar and set at pH 4.8. When required, the medium was supplemented with 10 mM uridine or 10 mM acetamide instead of (NH_4_)_2_SO_4_ (for *amdS* selection) or 5 μg/ml of chlorimuron ethyl (for acetolactate synthase *alS* gene selection) (Bower et al. 2012). *T. reesei* strains were routinely cultivated at 30 °C in the presence of light.

**Table 1:** Fungal strains and plasmids used in this study

	Genotype or description	Source or reference
Fungal strains		
QM6a	Wild-type strain	Anderson et al. 2013
P37∆cbhIpyrG-26	*pyr4* ^−^ and *Pcbh*I^−^	DuPont bioscience. Leiden, The Netherlands
JP7.7	P37∆cbhIpyrG-26 with I-*Sce*I restriction site cassette integrated at *cbh2* locus	This study
JP7.7_pTTT	JP7.7+ pTTT	This study
JP7.7.12	JP7.7+ pTTT-I*Sce*I	This study
JP7.7.14	JP7.7+ pTTT-I*Sce*I	This study
Plasmids		
pBJP6	Carries a I-*Sce*I restriction site cassette	This study
pCRpyrGAN	Contains the full gene of *A. nidulans pyrG*	This study
pTTT	*cbhI* promoter and *amdS* selection	DuPont bioscience
pTTT-ISceI	I-*Sce*I under control of the inducible *cbh1* promoter with *amdS* selection	This study
Ptrex6gGA/wt	Carries the *T. reesei* glucoamylase gene	DuPont bioscience
pJP8	Carries a *T. reesei* glucoamylase cassette with homologous regions of the I-*Sce*I landing sites cassette (*pBJP6*)	This study

*T. reesei* was transformed using the polyethylene glycol (PEG)-mediated protoplast transformation protocol described by Penttilä et al. (1987) with slight modifications. Fifty-millilitre cultures inoculated with 5 × 10^8^ conidia were grown in the dark at 30 °C and 200 rpm for 12–20 h. A total of 675 mg lysing enzyme (Sigma-Aldrich, Zwijndrecht, The Netherlands) were dissolved in 15 mL of 1.2 M MgSO_4_-10 mM sodium phosphate buffer, pH 5.8. Protoplasting was performed at 25 °C and 90 rpm and was verified every 30 min by microscopy. Two hundred microlitres of protoplast suspension was mixed with 5–10 μg of DNA and 2 mL of freshly made PEG buffer. Stable transformants were obtained by streaking on TrMM plates containing the required selection pressure, for two successive rounds. Single colonies obtained after double streaking were selected for sporulation and further analysis.

*T. reesei* liquid cultures were grown in 24-well plates configured such as to release lactose from a solid, porous matrix. Each well contained 1.25 mL of an NREL medium (9 g/L casamino acids, 5 g/L (NH_4_)_2_SO_4_, 4.5 g/L KH_2_PO4, 1 g/L MgSO_4_·7H_2_O, 1 g/L CaCl_2_·2H_2_O, 33 g/L PIPPS buffer, at pH 5.5, 0.25 % *T. reesei* trace elements, as described above). *Escherichia coli* DH5α strain was used for plasmid construction and propagation using standard techniques.

### DNA manipulations and molecular analyses

Genomic DNA extraction of *T. reesei*, diagnostic PCR and Southern blot analysis were performed as previously described (Meyer et al. 2010). Restriction enzymes and DNA dephosphorylation and ligation kits were obtained from Invitrogen (Bleiswijk, The Netherlands) or Thermo Fisher (Leusden, The Netherlands) and used according to the instructions of the manufacturer. Sequencing was performed by Macrogen (Amsterdam, The Netherlands).

### Construction of the I*-Sce*I restriction site cassette

The plasmid pBJP6 with two I-*Sce*I restriction sites was made as follows (Fig. S1 in the Supplementary Material). The 5′*UTR cbh2*, *PcbhI* and *Tcbh2* were amplified from genomic DNA of QM6a with primers GSP1 and GSP2, PP1 and PP2, and GSP3 and GSP4, respectively (Table 2). The PCR fragments were fused using Phusion DNA polymerase (Thermo Fisher, Leusden, The Netherlands) and cloned into *Xba*I and *Kpn*I sites of pBluescriptSK(+) (Stratagene, CA, USA) resulting in the plasmid pBJP4 (Fig. S1 in the Supplementary Material). DNA sequences encoding for N-terminally and C-terminally truncated green fluorescent protein (GFP) with 0.6-kbp overlapping sequences were synthetically ordered based on the *GFP* sequence of *Ptilosarcus* sp. (Geneart, Regensburg, Germany) and cloned into the *Nde*I site of pBJP4, resulting in the plasmid pBJP5. Restriction analysis was performed to ensure that the N-terminal sequence of GFP which contains the start codon is inserted properly after the promoter *cbhI*. The final pBPJ6 plasmid was obtained by amplifying the *pyrG* marker of *Aspergillus nidulans* flanked by two I-*Sce*I sites with the primers FwpGAN-ISceIPmeI and RevpGAN-ISceIPmeI from the plasmid pCRpyrGAN and cloned into the *Pme*I site of pBJP5. pCRpyrGAN is a pCR-Topo Blunt-based vector with the kanamycin resistance marker containing a PCR-amplified 1.7-kb genomic DNA fragment of the *A. nidulans pyrG* gene. Primers FwpGAN-ISceIPmeI and RevpGAN-ISceIPmeI were designed to incorporate the I-*Sce*I restriction sites and the *Pme*I sites downstream and upstream of the *pyrG* marker during PCR amplification (see primers sequences in Table 2). The sequence of the pBJP6 plasmid was confirmed by restriction analysis and sequencing.

**Table 2:** List of primers used in this study

Primer name	f/r	Sequence (5′ to 3′oriented)	Template
GSP1 (*Xba*I)	f	TCTAGAGGCTGTGCATTTCCGTTCTC	gDNA QM6a
GSP2	r	TGGTTACGGCAACAAACCTG	gDNA QM6a
PP1	f	CAGGTTTGTTGCCGTAACCAATTTGCCTGCTTGACCGACTG	gDNA QM6a
PP2	r	GGAACGATGGGTTTGCGTCCATATGGGGTAAGTCACTTACGGCAGC	gDNA QM6a
GSP3 (*Nde*I)	f	CCATATGGACGCAAACCCATCGTTCC	gDNA QM6a
GSP4 (*Kpn*I)	r	GGTACCGGTTCACCGCCTTATGTGAG	gDNA QM6a
FwpGAN-ISceI(*Pme*I)	f	GGTTTAAACCTAGGGATAACAGGGTAATTCGCCCTTGCTCTAGATAAC	pCRpyrGAN
RevpGAN-ISceI(*Pme*I)	r	GGTTTAAACCTAGGGATAACAGGGTAATAATTCGCCCTTGACTAGTGC	pCRpyrGAN
GSP5 (*Asi*SI)	f	GCGATCGCACGCAAACCCATCGTTCC	gDNA QM6a
GSP6 (*Asi*SI)	r	GCGATCGCGGTTCACCGCCTTATGTGAG	gDNA QM6a

Underlined sequences within the primers denote the I-*Sce*I restriction site

### Construction of the I*-Sce*I expression vector

A codon-optimized gene coding for the *Saccharomyces cerevisiae* I-*Sce*I sequence (SGD ID S000007279) was synthesized for expression in *T. reesei* (Geneart, Regensburg, Germany). The nucleotide sequence encoding I-*Sce*I has been deposited in GenBank under accession number KR584660. To construct a I-*Sce*I expression vector, the I-*Sce*I gene was cloned via a Gateway recombination into the telomeric plasmid pTTT (Aehle et al. 2011), where I-*Sce*I expression was driven by the *T. reesei cbhI*-inducible promoter. A I-*Sce*I variant in which we included the nuclear localization signal (NLS) (MATPSSVASS SSRDQVQRIH RVTRENRHLW YQLTVLQQPE RARACGSG) of the *T. reesei* Velvet protein (JGI ID 122284) was constructed and tested in parallel. To identify this putative NLS sequence in the *T. reesei* Velvet protein, the putative NLS sequence identified in the Velvet protein of *A. nidulans* (Stinnett et al. 2007) was used for alignments.

### Construction of the glucoamylase expression cassette for targeted integration

The plasmid pTrex6g-GA (Bower et al. 2012), which harboured the wild-type glucoamylase gene of *T. reesei* under control of the *cbhI* promoter, was used to construct the glucoamylase expression cassette for integration at the I-*Sce*I landing sites. To allow homologous integration of this reporter, the *cbh2* terminator region (*Tcbh2*) was cloned in pTrex6g-GA. The *Tcbh2* was amplified by PCR using the primers GSP5 and GSP6 (Table 2) and the genomic DNA of the *T. reesei* QM6a WT strain. The PCR product was digested with *Asi*SI and cloned into the same restriction sites of pTrex6g-GA, to form plasmid pJP8. The 10-kb glucoamylase expression fragment was cut out from pJP8 with *Psi*I and used for transformation.

### In vivo analysis of I*-Sce*I activity in *T. reesei* and fluorescent microscopy

Purified *T. reesei* transformants that carried I-*Sce*I restriction sites and the I-*Sce*I expression cassette were point inoculated on TrMM with 2 % of glucose or lactose as a carbon source with or without addition of uridine to the medium. The activity of I-*Sce*I was monitored by formation of sectors without growth after incubating transformants for several days at 30 °C. Sector formation is likely to be the consequence of excision of the *pyrG* marker and subsequent repair of the genomic DNA induced by the I-*Sce*I-mediated DSB. Transformants that formed sectors on solid medium were selected for GFP expression under fluorescence microscopy. A total of 10^7^ conidia of sector-forming transformants were inoculated in 5-mL germination medium containing 2 % glucose/sophorose (30:1) for induction and supplemented with 0.003 % yeast extract and 10 mM uridine, and cultivated on cover slips for 28 h at 30 °C. Samples were observed with Axioplan 2 fluorescence microscope (Zeiss, Sliedrecht, The Netherlands) equipped with a DKC-5000 digital camera (Sony) using different contrast or GFP settings. Images were captured and processed using Adobe Photoshop 6.0 (Adobe Systems Inc.).

### Quantification of the efficiency of *pyrG* excision mediated by I*-Sce*I expression

To determine the frequency of the *pyrG* marker loss as a consequence of I-*Sce*I expression, the strain JP7.7.12 bearing the I-*Sce*I restriction sites and the I-*Sce*I expressing cassette (pTTT-I*Sce*I) was used. As a control, strain JP7.7_pTTT bearing the I-*Sce*I restriction sites and the pTTT plasmid without I-*Sce*I was included. About 100 spores of each strain harvested from minimal medium without uridine were grown separately under inducing conditions (TrMM with 2 % lactose as carbon source) supplemented with uridine. Addition of uridine into the medium allowed for growth of all spores including those that will lose the *pyrG* marker under I-*Sce*I expression conditions. To quantify the frequency of the *pyrG* loss events, single colonies from I-*Sce*I-induced medium were transferred into minimal medium with and without uridine. The efficiency of the marker excision was determined as the ratio of the number of *pyrG-*negative colonies and the total number of colonies grown on the plate with uridine.

### Determination of glucoamylase activity and glucoamylase levels

Purified transformants were grown in production medium for 5 days and glucoamylase was measured from the culture filtrate. The activity of glucoamylase was determined using Betamyl as a substrate (Megazyme International, Bray, Ireland). Ten microlitres of the culture samples containing glucoamylase were mixed with 90 μL of Betamyl diluted in 50 mM sodium acetate pH 4.8. The reaction is performed at 37 °C for 20 min and quenched with 50 μL of 1 M sodium carbonate pH 9. Activity was monitored by the release of *p*-nitrophenol measured colorimetrically at 405 nm. For each transformant, the glucoamylase activity was measured in triplicate. Glucoamylase levels were detected using 10 % sodium dodecyl sulfate-polyacrylamide gel electrophoresis (SDS-PAGE) performed under denaturing conditions according to Laemmli (1970). Gels were stained with Coomassie Brilliant Blue R-250 (Bio-Rad, Veenendaal, The Netherlands).

## Results

### Design of a marker excision method to monitor I*-Sce*I activity

**Fig. 1:**
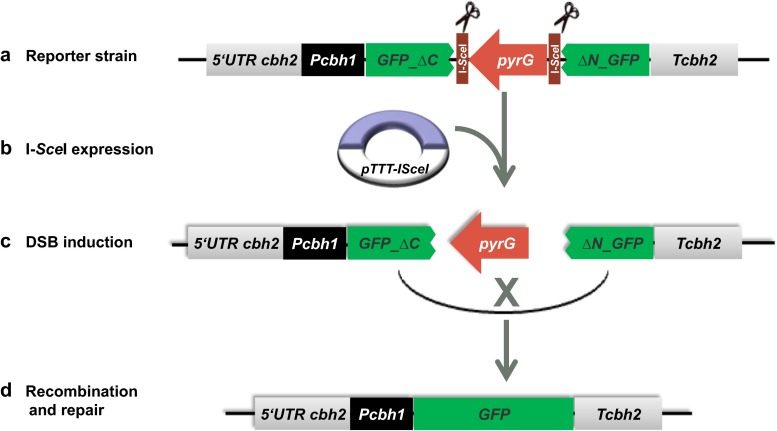
Schematic representation of the strategy to test the double-strand break (DSB) activity of I-*Sce*I expressed in *T. reesei*. The reporter cassette (pBJP6) with I-*Sce*I restriction sites inserted at the *cbh2* locus of a *T. reesei* uridine auxotrophic strain results in a uridine prototrophic strain containing two I-*Sce*I sites surrounding the *pyrG* cassette (*a*). Heterologous expression of I-*Sce*I is expected to generate two DSBs and the loss of the *pyrG* selection marker between the I-*Sce*I sites (*b*). The DSB can be repaired by homologous recombination between *GFP∆C* and *∆NGFP* adjacent to the breaks (*c*). After homologous recombination via the GFP direct repeats, this will result in a uridine auxotrophic strain (loss of the *pyrG* gene) and reconstitution of a functional GFP when correctly recombined via the direct repeat sequences (*d*). The resulting strain can be either screened for uridine auxotrophy or/and for GFP expression to monitor I-*Sce*I activity

With the aim to generate I-*Sce*I-mediated double-strand DNA breaks at a predetermined site in the *T. reesei* genome for targeted integration, we first wished to make sure that the expression of the *S. cerevisiae* gene encoding I-*Sce*I results in an active protein. To monitor the activity of the I-*Sce*I meganuclease in *T. reesei*, a reporter construct that contained two I-*Sce*I recognition sites flanking the *A. nidulans pyrG* selective marker was designed (Fig. 1). In addition, DNA with GFP direct repeats encoding an N-terminally (*ΔNGFP*) and C-terminally (*GFPΔC*) truncated, non-functional GFP were inserted around the I-*Sce*I-*pyrG-*I-*Sce*I sequence to provide regions for homologous recombination. Transcription of the *GFPΔC* part was regulated by the *cbhI* promoter. The pCbh1*-GFPΔC-*I-*Sce*I-*pyrG-*I-*Sce*I-*ΔNGFP* construct was subsequently cloned between ~1.5 kb 5′ and 3′ regions of the *cbh2* gene (0.6 kb in length) for targeted integration at the *cbh2* locus. The rationale of the strategy is that active I-*Sce*I expressed in a *T. reesei* strain containing this reporter construct would result in excision of the *pyrG* marker and repair of the double-strand break via the GFP repeats. A perfect repair would result in a uridine-auxotrophic strain expressing GFP (Fig. 1). It should be noted that the genome of *T. reesei* does not naturally contain a I-*Sce*I restriction site (5′-TAGGGATAACAGGGTAAT-3′).

### Construction of a *T. reesei* strain harbouring the I*-Sce*I restriction sites at the *cbh2* locus

The 7.5-kb reporter cassette as described above was linearized and introduced into a *T. reesei* strain deleted for *cbhI*. Transformants were analysed by Southern blot for correct integration at the *cbh2* locus. Southern blot analysis revealed three transformants harbouring a single copy of the complete I-*Sce*I cassette at the *cbh2* locus (JP7.7, JP7.9 and JP7.12) (Fig. S2 in the Supplementary Material). Other transformants either had multiple copies of the cassette integrated (JP7.10, JP7.11, JP7.13, JP7.14 and JP7.15) or did not seem to insert the complete cassette into the genome (JP7.8) (Fig. S2 in the Supplementary Material). Transformant JP7.7 was used in subsequent experiments.

### I*-Sce*I expression in *T. reesei* induces DSB at the targeted chromosomal locus

**Fig. 2:**
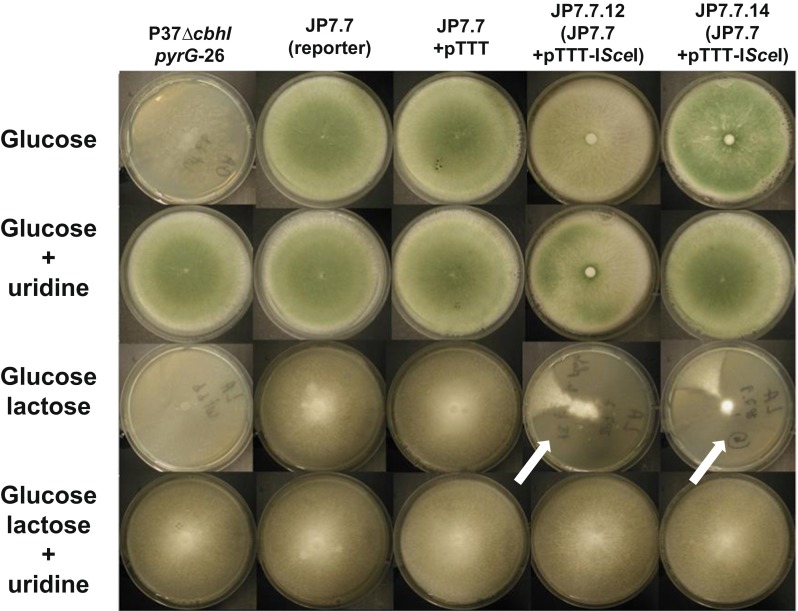
Loss of the *pyrG* cassette is mediated via I-*Sce*I meganuclease-induced loop-out recombination control strain (P37∆cbhIpyrG-26), and strains harbouring the I-*Sce*I restriction site-containing construct (JP7.7 transformants) were transformed with either the control plasmid (pTTT) or the I-*Sce*I expression plasmid (pTTT-I*Sce*I). Spores of the strains were point-inoculated in the centre of a 9-cm Petri dish containing minimal medium and containing either 50 mM glucose (glucose) or a mix of 50 mM glucose and 50 mM lactose (glucose and lactose) either with or without the addition of uridine. Pictures of the colonies were taken after 4 days of growth at 30 °C. Induction of I-*Sce*I expression by lactose in JP7.7 transformants containing the pTTT-I*Sce*I vector results in sectored colonies. These sectors are regions of no growth expected to be caused by loop out of the *pyrG* marker and the subsequent inability of the strain to grow on the medium without uridine. Sectoring is dependent on the expression of I-*Sce*I via lactose induction and no longer visible if uridine is supplemented. *Arrows* indicate regions with no growth. As expected, the parental strain does not grow on media without uridine

A codon-optimized version of I-*Sce*I with or without an additional NLS sequence from the *T. reesei vel1* gene was introduced on a replicative plasmid into JP7.7 strain bearing the I-*Sce*I landing sites. Expression of I-*Sce*I nuclease was controlled by the *cbh1* promoter, which is repressed by glucose and strongly induced by lactose or sophorose (Ivanova et al. 2013; Xu et al. 2014). Transformants were grown either on glucose- or lactose/glucose-containing medium. As shown in Fig. 2, induction by lactose in the strains expressing I-*Sce*I and containing the I-*Sce*I reporter cassette resulted in sectored colonies. This sectoring could be interpreted as I-*Sce*I activity generating a double-strand break, which was repaired via recombination between the GFP direct repeats leading to loss of the *pyrG* marker. As a consequence of efficient recombination in all nuclei in some part of the colony, this part of the colony no longer grew on plates without uridine giving rise to a non-growing sector. As shown in Fig. 2, sectors were present when lactose was used as a carbon source, although we occasionally (less than 10 % of the glucose/lactose plates) also observed sectors on glucose plates indicating that some I-*Sce*I might be expressed (not shown). No sectors were observed in the control strains (JP7.7) or JP7.7 containing the empty vector (JP7.7+ pTTT), indicating the *Sce*I expression is required for looping out the *pyrG* marker. Similar sectoring on lactose was observed for transformants expressing I-*Sce*I with NLS_vel1_ but never for the control strains (Fig. 2 and data not shown). When uridine was added to the plates, non-growing sectors were absent indicating that they were caused by loss of the *pyrG* marker (Fig. 2). These results strongly suggest that the *S. cerevisiae* I-*Sce*I was expressed and active in *T. reesei*.

**Fig. 3:**
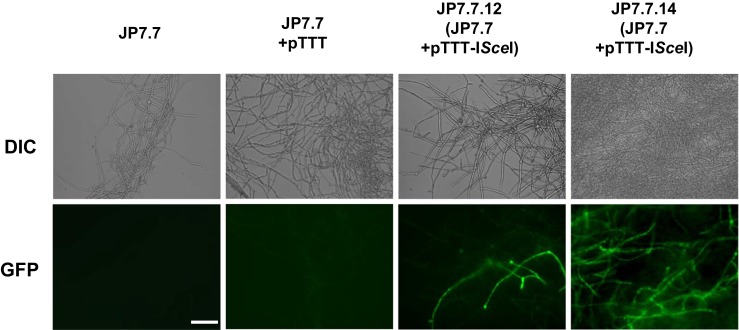
Visualization of GFP fluorescence after I-*Sce*I meganuclease-mediated loop-out recombination. Induction of I-*Sce*I expression by sophorose results in excision of the *pyrG* marker and reconstitution of a functional GFP, indicated by the green fluorescent hyphae. Strains were grown on TrMM containing 2 % glucose/sophorose (30:1) and uridine to induce I-*Sce*I expression and allow the loss of the *pyrG* marker, respectively. Strain JP7.7, harbouring I-*Sce*I restriction sites but not expressing the I-*Sce*I gene, was included in the analysis. Lack of GFP expression indicates that the loop-out event to restore GFP functionality requires I-*Sce*I. *Bars*, 10 μm

Functionality of I-*Sce*I was further confirmed by fluorescent microscopy in liquid cultures. Hyphae grown in the presence of sophorose showed green fluorescence indicating reconstitution of a functional GFP as a consequence of the *pyrG* excision followed by a recombination event. As GFP was observed only in strains expressing I-*Sce*I, this further indicates that I-*Sce*I was actively expressed (Fig. 3).

The frequency of the I-*Sce*I-mediated excision was determined by comparing the frequency of losing the *pyrG* marker in the I-*Sce*I-expressing and I-*Sce*I-non-expressing strains (strains JP7.7.12 and JP7.7_pTTT, respectively). Therefore, about 100 spores of each strain were plated on lactose-containing induction medium supplemented with uridine. After growth for 3 days, each colony formed was further scored for its growth in the absence of uridine. Sixty-six percent of the colonies of the JP7.7.12 strain bearing the I-*Sce*I restriction sites and containing the I-*Sce*I expression cassette (*pTTT-*I*Sce*I) were found to be auxotrophic for uridine, whereas only 1.3 % of the colonies of JP7.7_pTTT strain bearing the empty pTTT plasmid were uridine-dependent. Thus, a high frequency of *pyrG* excision mediated by in vivo-expressed I-*Sce*I nuclease was observed during a plate growth test.

### I*-Sce*I-mediated DSB increases transformation efficiency and transformant stability

**Fig. 4:**
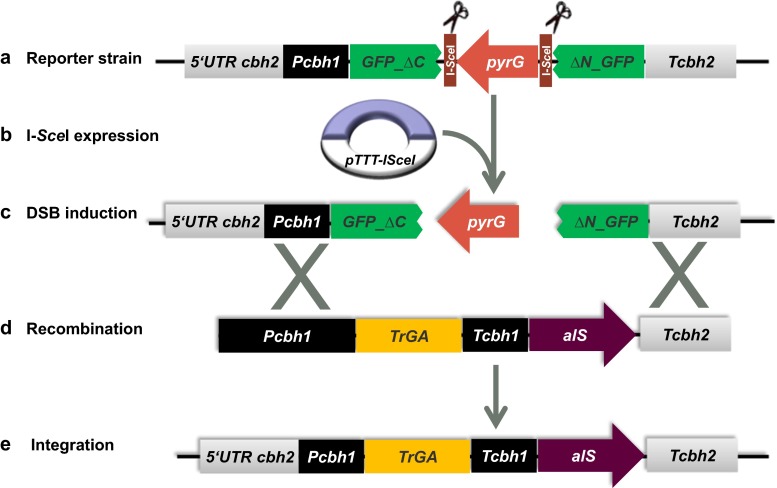
Schematic representation of targeted integration of the glucoamylase expression cassette via I-*Sce*I-mediated homologous recombination. (*a*) As a starting strain, the same reporter strain (JP7.7) was used as before. This strain contains the cassette with the I-*Sce*I restriction sites inserted at the *cbh2* locus of *T. reesei*. (*b*) Transformation of JP7.7 with the vector to express the I-*Sce*I gene (pTTT-I*Sce*I). This strain can be propagated on glucose medium without uridine to keep selection pressure on maintaining the *pyrG* cassette. (*c* and *d*) Transformation of the glucoamylase-expressing cassette (pJP8) and simultaneous induction of I-*Sce*I expression to create a DSB. I-*Sce*I was induced by plating out protoplasts on lactose-containing transformation plates. The DSB can be repaired by homologous recombination with the glucoamylase-expressing cassette (pJP8), which has homology regions to the locus containing the I-*Sce*I sites. (e) Targeted integration of the glucoamylase cassette at the I-*Sce*I landing site would generate a strain that is resistant to chlorimuron ethyl (*alS* ^*+*^) and uridine auxotrophic (*pyrG* ^*−*^) and is expected to contain the glucoamylase gene under control of the *cbhI* promoter

**Fig. 5:**
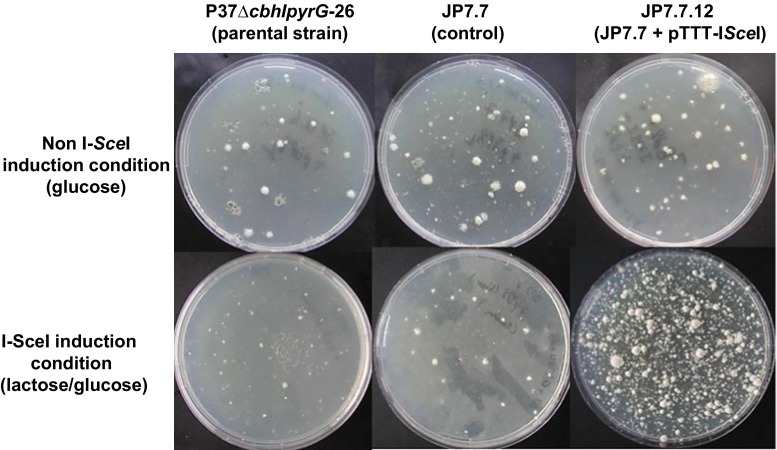
Transformation under I-*Sce*I-inducing and non-inducing conditions. The glucoamylase-expressing cassette (pJP8) with homologous regions to the locus containing the I-*Sce*I restriction sites was transformed into the parental strain (P37*)*, JP7.7 (containing the I-*Sce*I restriction site construct) and JP7.7.12 (containing the I-*Sce*I restriction site construct and expressing I-*Sce*I*)*, respectively, by PEG transformation. The protoplasts were plated out on glucose- or lactose/glucose-based transformation medium supplemented with chlorimuron ethyl and uridine. Transformants were photographed after 3 days of incubation

To determine whether a DSB mediated by I-*Sce*I can increase transformation and recombination efficiency in *T. reesei*, the aforementioned reporter strain JP7.7.12 was transformed with a linear fragment comprising a glucoamylase expression cassette (Fig. 4). This cassette contains the *alS* selection marker which renders transformations to become resistant against chlorimuron ethyl (Bower et al. 2012). Chlorimuron ethyl-resistant transformants were selected either on glucose (I-*Sce*I-repressed conditions) or lactose- and glucose-containing medium (I-*Sce*I-inducing condition). As a control, we also transformed a strain carrying only the I-*Sce*I restriction sites (JP7.7) with the same DNA fragment. As shown in Fig. 5 and Table 3, I-*Sce*I induction increased the number of chlorimuron ethyl-resistant transformants more than threefold and sixfold as compared to repressed conditions and to the control, respectively.

**Table 3:** The effect of I-*Sce*I induction on number and stability of transformants

Strain	C source	# of primary transformants^a^	% stable transformants^b^	% Gla-positive transformants^c^	% Gla-positive pyrG ^−^ transformants^d^	Homologous recombination efficiency^e^
JP7.7 (Control)	Lactose	32	17/32 (53 %)	Not tested	Not tested	Not tested
JP7.7 (Control)	Glucose	36	21/36 (58 %)	12/40 (30 %)	2/12 (16 %)	2/12 (16 %)
JP7.7.12 (pTTT-ISceI)	Lactose	>226	45/50 (90 %)	22/40 (55 %)	15/22 (68 %)	15/22 (68 %)
JP7.7.12 (pTTT-ISceI)	Glucose	70	22/50 (44 %)	15/40 (37 %)	7/15 (46 %)	7/15 (46 %)

^a^Number of primary transformants on transformation plates (TrMMsorb + uridine + chlorimuron ethyl (Als substrate)) containing 1 % of lactose and 1 % glucose referred to as induced and non-induced expression of I-*Sce*I, respectively. Control transformations have been carried out using the transformation medium using the parental strain JP7.7.

^b^Primary transformants were purified on TrMM + uridine + chlorimuron ethyl. The stability of the transformants is defined by their ability to grow in selective TrMM after double purification. Stable transformants grow well on these selective plates. Abortive transformants (not stable) do not grow

^c^Stable transformants were tested for glucoamylase activity in a microtitre-based growth/activity assay. Transformants showing Gla activity above the background were considered as Gla-positive transformants

^d^Gla-positive transformants were tested for the *pyrG* phenotype by inoculating spores of each transformant on TrMM with uridine or TrMM without uridine. Strains were considered *pyrG* minus (uridine auxotroph) when growing on TrMM + uridine, but not growing om TrMM without uridine

^e^GlaA expressing and *pyrG* minus strain were analysed by Southern blot and confirmed an integration pattern indicative of homologous integration of the *glaA* expression cassette at the I-*Sce*I landing site

We further analysed the stability of the transformants obtained in each group of transformation, as by subsequent purification on MM-chlorimuron ethyl plates. Ninety percent of the transformants obtained after the induction of I-*Sce*I were stable against 44 and 58 % of colonies grown under repressed conditions and for the control strain, respectively (Table 3). This indicates that heterologous expression of the I-*Sce*I nuclease increased transformation efficacy and transformant stability. Instability of transformants usually results from a lack of integration of a given DNA cassette into the genome and has been previously described to be a major bottleneck for *T. reesei* transformation (Jørgensen et al. 2014).

**Fig. 6:**
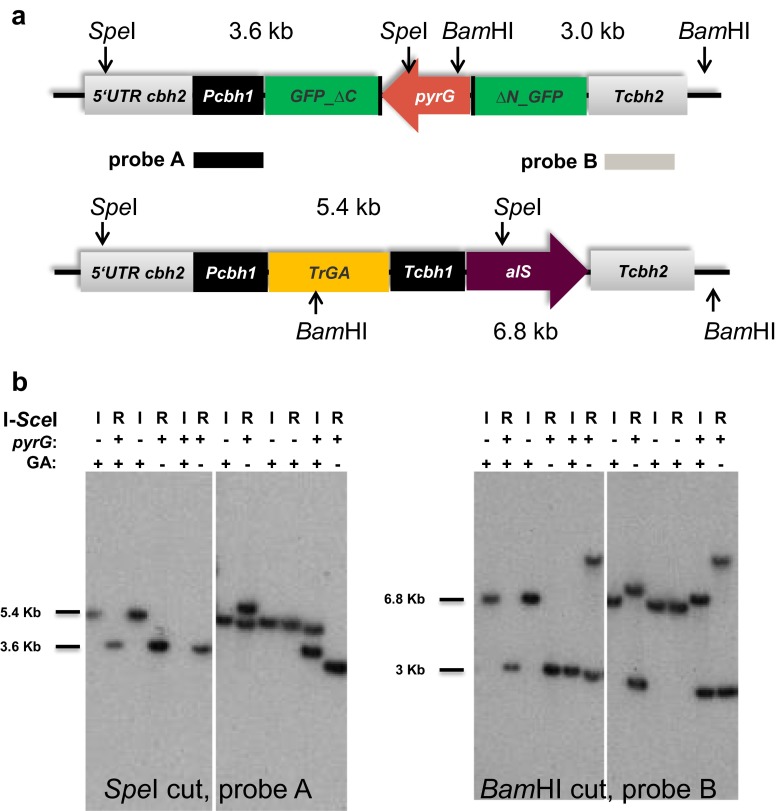
Southern blot analysis of selected transformants after transformation with the glucoamylase expression cassette. **a** Diagram of pJP8 transformants to analyse integration of the glucoamylase cassette at the 5′ flank and 3′ flank of the *cbh2* locus by using *Spe*I and *Bam*HI as restriction enzymes and *PcbhI* and *Tcbh2* as probes, respectively. The expected band size is 5.4 kb for integration of the glucoamylase cassette using the *PcbhI* probe and 6.8 kb for integration using the *Tcbh2* probe. A non-homologous integration of the glucoamylase cassette will not alter the c*bh2* locus and a hybridizing DNA fragment of 3.6 and 3 kb are expected with the *PcbhI* probe and the c*bh2* probe, respectively. **b** Southern blot results of pJP8 transformants to analyse the integration of the glucoamylase cassette at the 5′ flank (*left blot*) and 3′ flank (*right blot*) of the *cbh2.* The two blots shown for analysis of the 5′ flank integration or 3′ flank integration are from a single Southern blot. Some lanes of the blot have been removed to exclude unrestricted genomic DNA samples. *I* I-*Sce*I is induced, *R* I-*Sce*I is repressed, + indicate presence of *pyrG* marker or glucoamylase expression, − indicate absence of the *pyrG* marker or non-expression of glucoamylase

Targeted integration of the glucoamylase expression cassette at the predetermined genomic locus would result in acquisition of chlorimuron ethyl resistance (*alS*^+^ phenotype), uridine auxotrophy, caused by a loss of the *pyrG* marker, and the presence of the glucoamylase expression cassette (Fig. 4). To determine whether the I-*Sce*I-mediated targeted integration correlated with the expression of the introduced glucoamylase cassette, we assayed ~40 stable transformants from each transformation group for glucoamylase activity and screened the glucoamylase-positive transformants for their *pyrG* phenotype. As shown in Table 3, about two thirds of the glucoamylase-positive transformants (15 of 22) obtained upon I-*Sce*I induction became uridine-auxotrophic, indicating that the construct was integrated at the intended locus via a double-crossover event, thereby removing the *pyrG* marker. Frequencies of homologous recombination were ~46 and ~16 % under I-*Sce*I repression and in the control strain, respectively. These results show that expression of I-*Sce*I increased both the transformation efficiency and the frequency of targeted integration in *T. reesei.* Leaky expression from the *cbhI* promoter in glucose medium could explain the increased number of targeted integration transformants under repressed conditions, as compared to the control transformants. Overall analysis showed that ~93 % (14/15) uridine-requiring strains expressed glucoamylase when I-*Sce*I was induced, thereby confirming strong correlation between a *pyrG*-negative phenotype and glucoamylase expression. We further analysed genomic DNA from selected transformants for the integration pattern of the glucoamylase cassette by Southern blot. The uridine-auxotrophic transformants expressing glucoamylase contained the complete glucoamylase cassette integrated in a single copy at the predetermined *cbh2* locus (Fig. 6a, b). *PyrG*^*+*^ transformants not showing glucoamylase activity very likely did not or only partially integrated the glucoamylase cassette. *PyrG*^*+*^ transformants displaying glucoamylase activity resulted probably from a partial integration of the cassette with the intact glucoamylase gene into the genome (Fig. 6a, b; PCR, data not shown).

### Uniform expression of the reporter glucoamylase gene after targeted integration at the engineered genomic locus

**Fig. 7:**
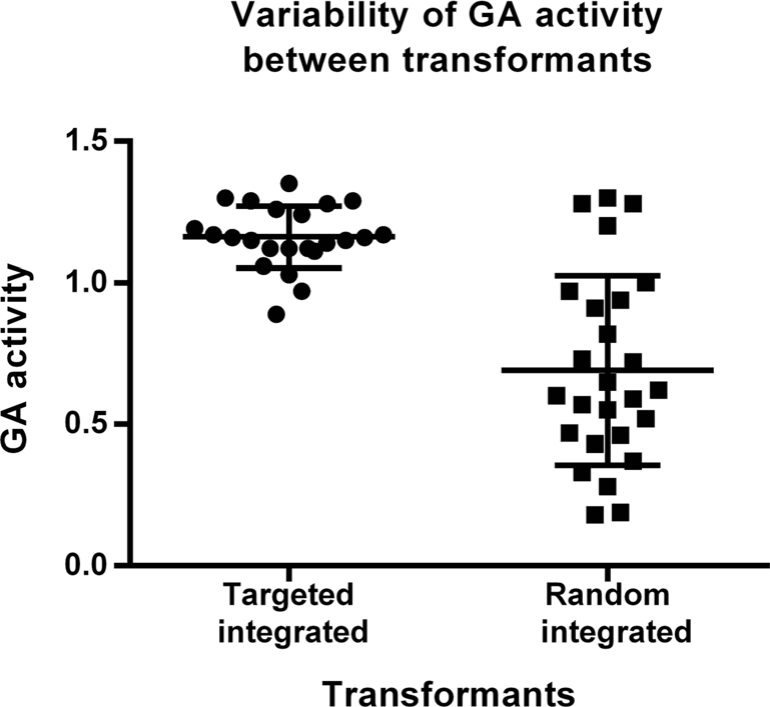
Glucoamylase activity in culture medium of transformants obtained after targeted or random integration. Targeted integrated transformants displayed high homogeneity of glucoamylase activity compared to transformants with random integration of the glucoamylase cassette. The analysed transformants were tested based on their auxotrophy to uridine. The targeted (*n* = 23) and random (*n* = 26) integrated transformants are *pyrG* ^*−*^ and *pyrG* ^*+*^ phenotype, respectively. The *pyrG* ^*−*^ transformants were further characterized for correct targeting of the glucoamylase cassette at the *cbh2* locus. Plots were created using GraphPad Prism 6 (column scatter graph). The *horizontal bars* represent the mean values with standard deviations

To compare the variability in glucoamylase (GA) expression among transformants with targeted integration of the GA cassette (*pyrG*^−^ transformants) versus random integrated transformants (*pyrG*^+^ transformants), strains were grown in sophorose-containing medium to induce GA, and culture samples were assayed for glucoamylase activity. As shown in Fig. 7, *pyrG*^*−*^ transformants obtained displayed a more consistent pattern of glucoamylase activity as compared to the expression profile of *pyrG*^+^ transformants. Nearly 80 % of transformant GA activity varied within a range of 15–20 %, which could be explained by a single-copy integration of the GA fragment at the engineered locus. The variation observed resulted from fermentation conditions rather than differences in the genetic background of the transformants. In contrast, a random integration of the expression cassette gave rise to significant variation in GA levels among transformants (Fig. 7). On average, the transformant obtained by random integration displayed lower glucoamylase activity than the transformant obtained by targeted integration and could be due to the targeting of the expression cassette to the *cbh2* locus, which is likely to be an actively transcribed region with a positive effect on glucoamylase production which is driven by the *cbh1* promoter.

## Discussion

Rare-cutting, double-stranded DNA endonucleases, called meganucleases, have emerged as a powerful tool for genome manipulation. Several studies have demonstrated that expression of such endonucleases in prokaryotic and eukaryotic cells stimulated homologous recombination between a given repair template and a genome locus containing an endonuclease recognition site (Choulika et al. 1995; Glover and Horn 2009; Meddows et al. 2005; Rouet et al. 1994). The aim of the current study was to increase efficiency of the gene-targeted integration at a predetermined locus in the *T. reesei* genome to ensure high expression levels and limited variability of protein expression. To this end, the *S. cerevisiae* endonuclease I-*Sce*I was expressed in *T. reesei* and I-*Sce*I recognition sites flanking a reporter construct were introduced into the *cbh2* locus of *T. reesei*. We demonstrated that expression of I-*Sce*I resulted in endonuclease activity. The I-*Sce*I protein appeared to be imported into the *T. reesei* nucleus via the intrinsic NLS signal, as we did not detect obvious differences in efficiency of marker excision between the original I-*Sce*I protein and a I-*Sce*I variant fused to a nuclear targeting signal of the *T. reesei* Vel1 protein. We analysed the impact of the I-*Sce*I-mediated DSBs on transformation frequency, transformant stability and efficiency of homologous recombination.

Our data indicate that DSB mediated by I-*Sce*I in *T. reesei* improves the efficiency of transformation and the stability of the transformants and promotes gene targeting at a defined locus. At least sixfold more transformants were obtained when I-*Sce*I was induced, and, more importantly, 90 % of them were found to be stable. Genetic instability is a major bottleneck in *T. reesei* transformation and usually is a consequence of a lack of integration of a transformed DNA cassette into the genome or due to a tandem integration of multiple copies, which could be excised through a loop-out event (Aw and Polizzi 2013; Jørgensen et al. 2014; Le Dall et al. 1994; Lee and Da Silva 1997; Ohi et al. 1998). This is the first study that addresses issues related to the stability of transformants mediated by I-*Sce*I in filamentous fungi and particularly in the industrial fungus *T. reesei*. Furthermore, when I-*Sce*I was expressed in the cells, the frequency of homologous recombination increased up to 68 %.

A recent study of gene targeting based on I-*Sce*I-induced DSBs in *P. oryzae* (*Magnaporthe oryzae*) reported that in this fungus, I-*Sce*I expression increases targeted integration up to about 40 %, which is comparable to our findings (Arazoe et al. 2014). Targeted integration can be also improved in filamentous fungi by deletion of the genes involved in non-homologous end joined (NHEJ) recombination (Ninomiya et al. 2004; Krappmann et al. 2006; Meyer et al. 2007; Guangtao et al. 2009; Steiger et al. 2011). However, inactivation of the NHEJ genes usually increases strain sensitivity towards chemicals and physical DNA-damaging agents and mostly reduces efficiency of transformation as compared to a strain with intact NHEJ genes (Zhang et al. 2011). In addition, targeted integration in strains deficient of NHEJ proteins can be highly locus-dependent and may vary from 33 to 100 % depending on the insertion site (Jørgensen et al. 2014; Schuster et al. 2012). Our study shows that using the I-*Sce*I endonuclease, we can improve targeted integration events in a *T. reesei* strain with intact NHEJ genes. Moreover, the targeted integration transformants could be specifically selected by adding 5-fluoroorotic acid (5′FOA) in the transformation medium. About 90 % of the *pyrG*^−^ transformants expressed glucoamylase, indicating that the DNA cassette was integrated at the intended locus. Moreover, Southern blot analysis confirmed the presence of a single copy of the expression fragment in these transformants. Importantly, we found that glucoamylase production levels were much more consistent among the transformants that harboured the expression cassette at the targeted locus as compared to transformants with a random integration pattern. Homogeneity in protein expression in the population of transformants is always a great challenge in the field of fungal biotechnology because of the low efficiency of targeted integration events (Aw and Polizzi 2013). Thus, our technique may be employed for high-throughput screening of enzyme libraries or for construction of production strains with predetermined integration sites, which is especially relevant in industrial biotechnology.

## Electronic supplementary materials

ESM 1 (PDF 294 kb)
