# Supplementary material for: I-SceI-mediated double-strand DNA breaks stimulate efficient gene targeting in the industrial fungus Trichoderma reesei
Source: Appl Microbiol Biotechnol. 2015 Aug 15;99(23):10083–95. doi: 10.1007/s00253-015-6829-1 (PMC4643118; doi:10.1007/s00253-015-6829-1)
Supplement: Supplementary file 1 — (PDF 294 kb) [file 253_2015_6829_MOESM1_ESM.pdf]

Journal Name: Applied Microbiology and Biotechnology

Manuscript Title: I-*Sce*I-mediated double-strand DNA breaks stimulate efficient gene targeting in the industrial fungus *Trichoderma reesei*

Jean Paul Ouedraogo[1], Mark Arentshorst[1], Igor Nikolaev[2], Sharief Barends[2] and Arthur F.J. Ram[1*]

[1] Molecular Microbiology and Biotechnology, Institute of Biology Leiden, Kluyver Centre for Genomics of Industrial Fermentation, Leiden University, Sylviusweg 72, 2333 BE Leiden, The Netherlands

[2] Dupont Industrial Biosciences, Archimedesweg 30, 2333 CN Leiden, The Netherlan

*Corresponding author: Arthur F.J. Ram

e-mail: A.F.J.Ram@biology.leidenuniv.nl

Tel: +31 (0)71 5274914

Fax: +31 (0)71 5274999

Fig. S1

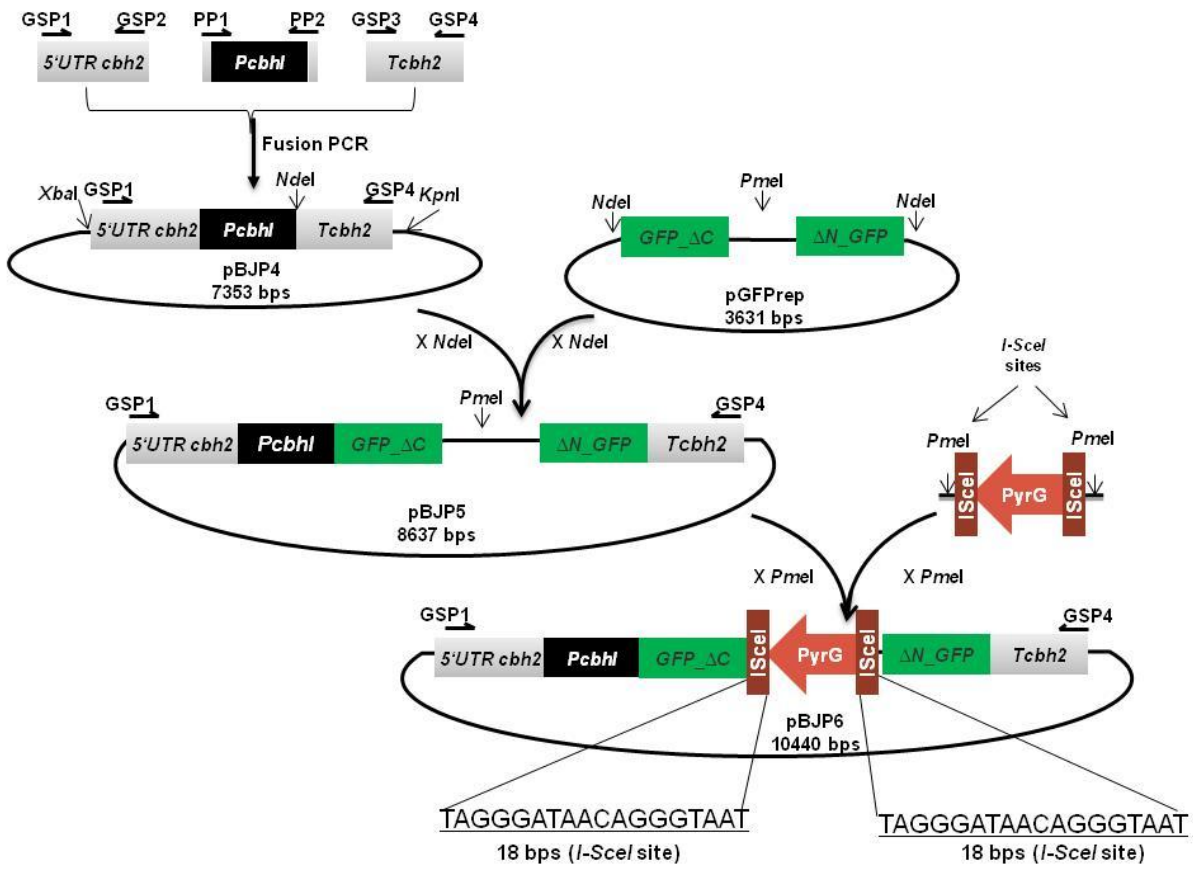

Fig. S1 Construction of plasmid pBJP6 containing the I-*Sce*I restriction sites. ~1.5 kb 5' UTR *cbh2*, *PcbhI* and *Tcbh2* were fused together by fusion PCR and cloned into *Xba*I and *Kpn*I sites of pBluescript SK(+) to generate the 7.3-kb pBJP4 plasmid. A synthetic direct repeat sequence of truncated GFP was inserted into the single *Nde*I restriction site of pBJP4, while correct orientation with *GFPΔC* inserted after the promoter *cbhI* lead to plasmid pBJP5 (8.6 Kb). The final 10-kb pBJP6 plasmid with I-*Sce*I restriction sites was obtained by cloning the PCR-amplified *A. nidulans pyrG* marker surrounded by two I-*Sce*I restriction site into the single *Pme*I site of pBJP5.

Fig. S2

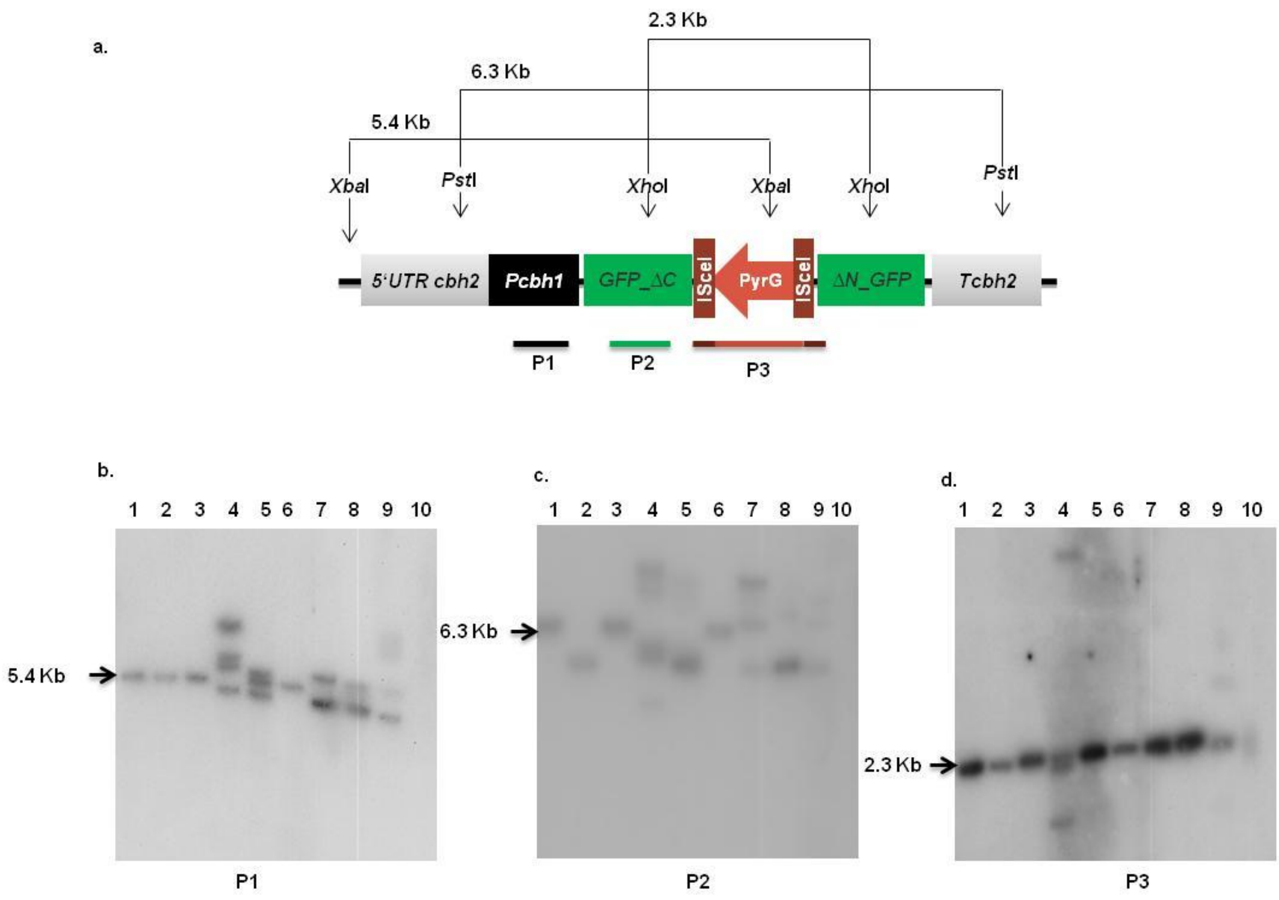

Fig. S2 Southern blot analysis of transformants containing the I-*Sce*I restriction sites in the genome of *T. reesei* P37Δ*cbhIpyrG*-26. (a) Detailed diagram of the I-*Sce*I restriction sites cassette after expected homologous recombination in the genome of *T. reesei*. The restriction enzymes used to perform the Southern blot are indicated by arrows. Three different probes (P1, P2, P3) represented by colour bars are used to confirm the presence of the complete construct and the copy number. The size of the expected band is indicated on the left. (b) Southern blot analysis of nine transformants (lane 1 - 9 represent JP7.7, JP7.8, JP7.9, JP7.10, JP7.11, JP7.12, JP7.13, JP7.14, and JP7.15, respectively) and the parental strain P37Δ*cbhIpyrG*-26 (lane 10). Genomic DNA of the strains was digested with *Xba*I and then hybridized with probe P1. The 5.4-kb band size represents the I-*Sce*I restriction cassette inserted in the genome. (c) Genomic DNA was digested with *Pst*I and hybridized with probe P2. The expected band size of the integrated I-*Sce*I cassette is 6.3 kb. (d) Genomic DNA was digested with *Xho*I and hybridized with probe P3. The expected band size with probe P3 is 2.3 kb.
